# Supplementary material for: Integrated silicon qubit platform with single-spin addressability, exchange control and single-shot singlet-triplet readout
Source: Nat Commun. 2018 Oct 30;9:4370. doi: 10.1038/s41467-018-06039-x (PMC6207676; doi:10.1038/s41467-018-06039-x)
Supplement: Supplementary file 1 — Supplementary Information [file 41467_2018_6039_MOESM1_ESM.pdf]

Supplementary Information

Integrated silicon qubit platform with single-spin addressability, exchange control and single-shot singlet-triplet readout

Fogarty et al.

SUPPLEMENTARY FIGURES

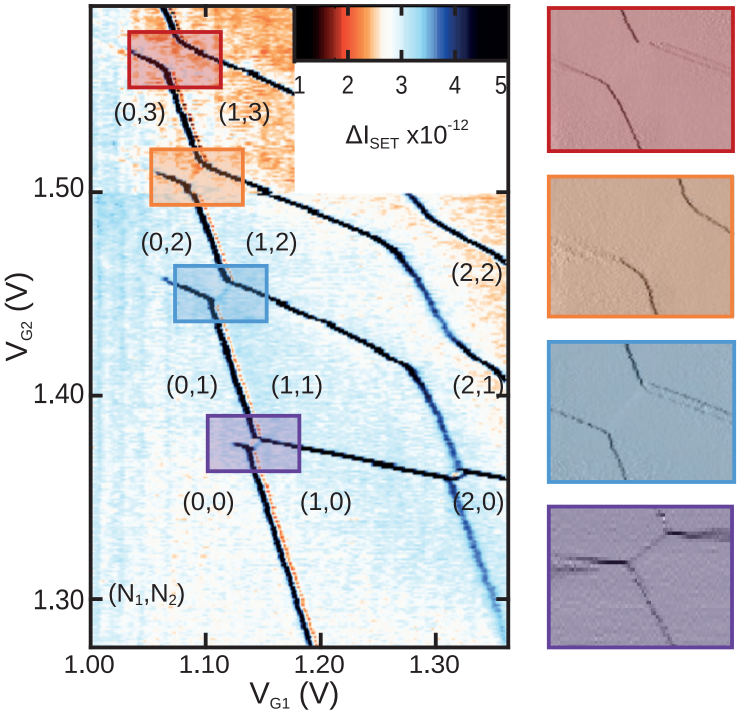

Supplementary Figure 1. | **Anti-crossing dependent latching** Charge stability diagram (left) of the double-dot system. Mapping (as described in the main text) at anti-crossing $(0, N_2 + 1) - (1, N_2)$ for $N_2 = 0 - 3$, shown (right) for a sequence which involves a random loading of the $(1, N_2)$ state. The latched readout is observed only at every second anti-crossing (when N_2 is odd), consistent with mapping of the PSB charge states to a dot-reservoir charge state

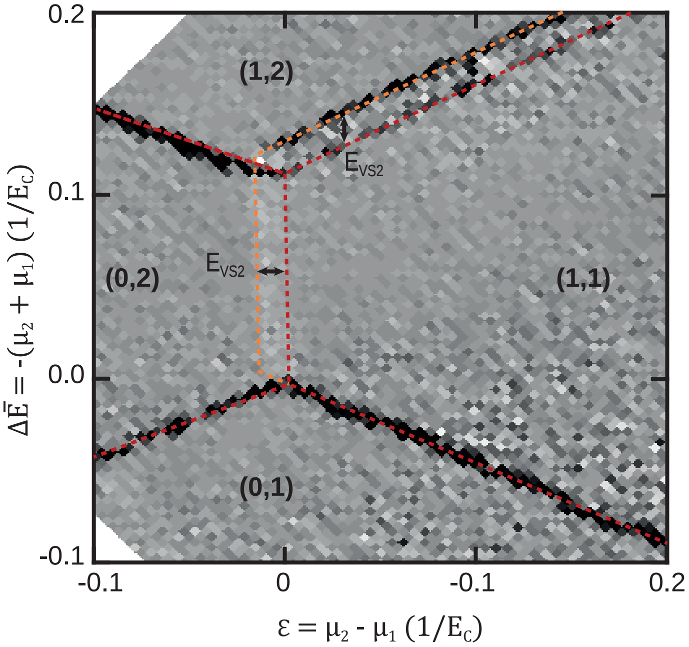

Supplementary Figure 2. | **Double dot chemical potential and valley splitting.** Transformation of three level pulse sequence from Fig. 1d to illustrate detuning ε and average energy $\bar{E}$. Valley splitting for dot $G2$ can be measured with respect to charging energy E_{C} via the splitting of standard-PSB and Latched-PSB.

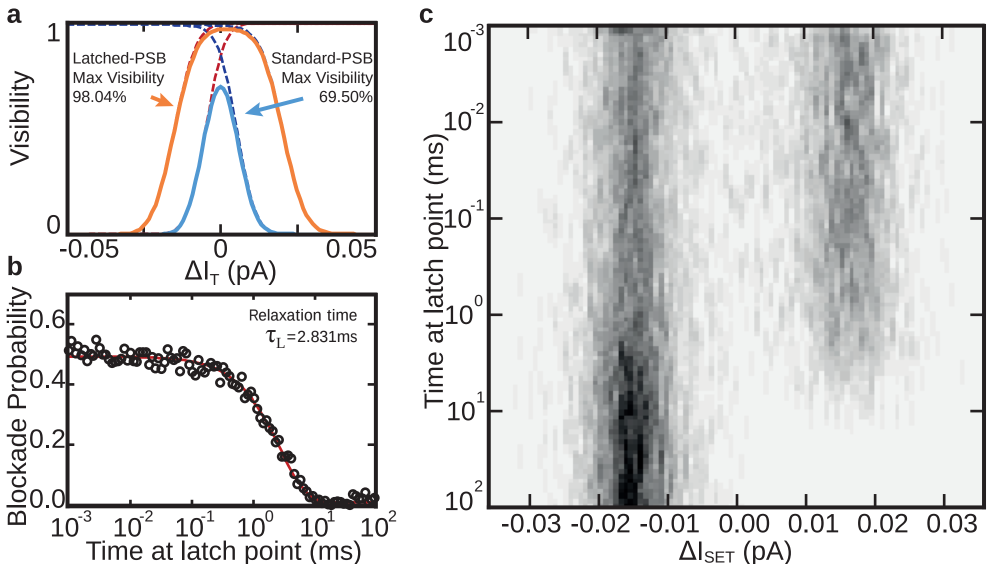

Supplementary Figure 3. | **Analysis of latched readout.** **a)** Maximum fidelity with optimized threshold voltage as given by process described in Ref.[1]. Dashed curves indicate the measurement fidelity of the singlet (red) and triplet (blue) signals. **b)** Latched-PSB readout lifetime measurement as performed by intitalizing random (1,1) state, pulsing rapidly to $(0, 2)$ point (Fig. 1f), then to latched point (Fig. 1g) to better emulate S/T experiments. Here, the characteristic time is given by latched lifetime τ_L. The error rate due to misidentification is reduced by 93.57% via latched readout to a maximum visibility of 98%. A visibility decrease to approximately 75% is observed when crossing from (1,1) to (0,2) charge configuration before pulsing to the (1,2) readout point. **c)** Histograms of the latched readout as a function of dwell time at the readout position.

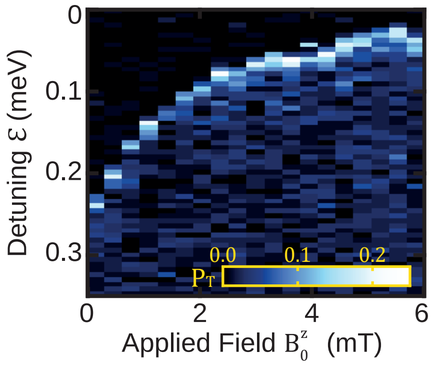

Supplementary Figure 4. | **Spin Funnel Data.** The spin funnel used for data collation in Fig. 4 of the main text. Data is taken for the same device cooldown.

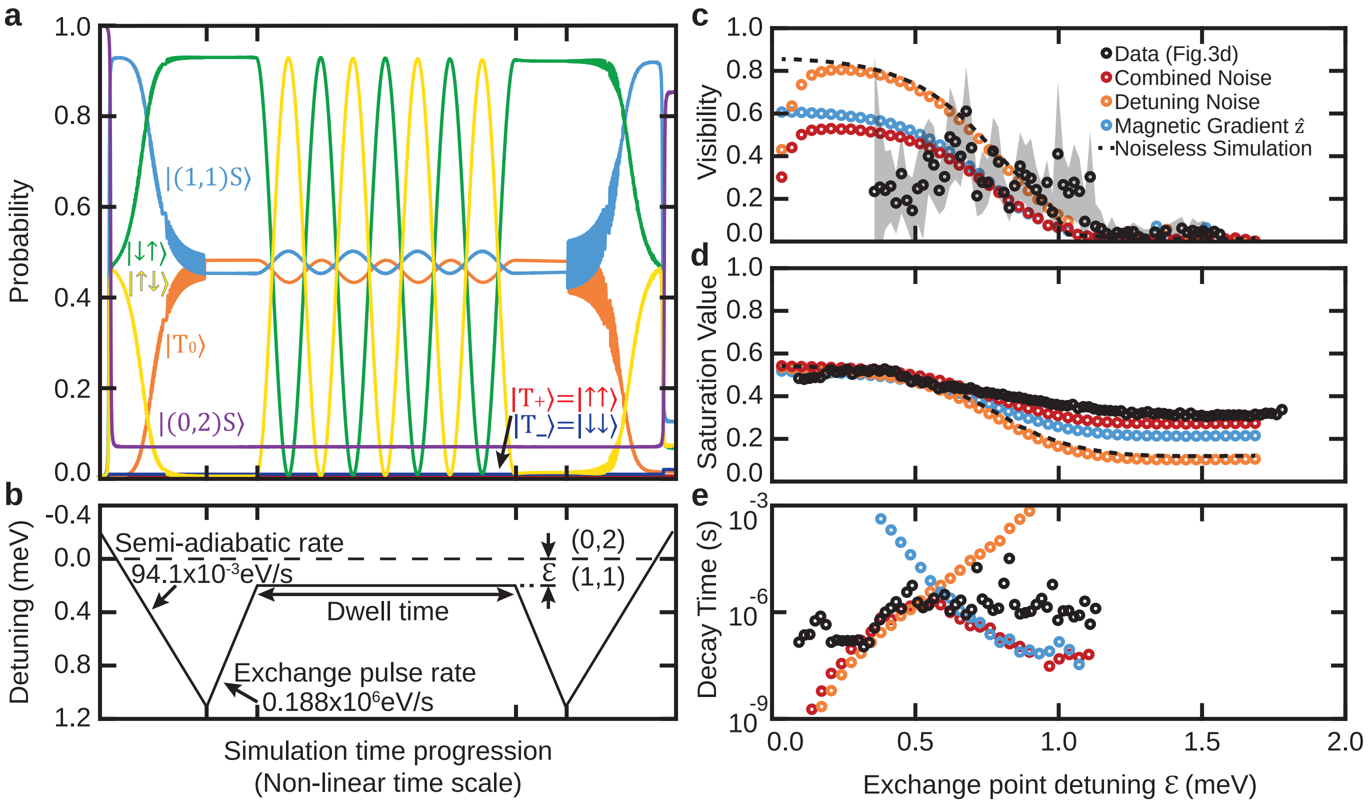

Supplementary Figure 5. | **Numerical simulation of exchange oscillations under realistic noise. a)** Simulation example illustrating the combination of all preparation and measurement errors including state leakages. The process involves transfer of a $|(0,2)S\rangle$ state to $|(1,1)S\rangle$, followed by semi-adiabatic transfer to $|\downarrow,\uparrow\rangle$, pulse to the exchange point for a dwell time, followed by reversal of the exchange pulse and semi-adiabatic mapping. **b)** Shows the detuning pulse sequence for a) against simulation time steps (non-linear time progression for illustrative purposes). Simulation time-steps are normalized to ramping speeds for each individual pulse region. **c-e)** Shows fitting parameters from Supplementary Eq. 6 after time evolution at the exchange point ε. Comparisons are drawn between the separate contributions of the most detrimental noise couplings from Supplementary Table 1 and a cumulative noise of all mechanisms. **c)** Upper bound visibility metric V_{Sig} from Supplementary Eq. 6 for the experimental data compared against the same visibility metric produced from simulations involving electric and magnetic field fluctuations. **d)** Illustrates the blockade saturation value V_{Sat}, which can indicate e_{Transfer} and e_{Map} error processes. **e)** Fitting parameter for driven oscillation decay time T_2^{ρ} produced from frequency variation σ_F (see text) for both the experimental data and the simulations.

Noise Type	Hamiltonian Parameter	Governing noise source
Exchange noise	$J(\varepsilon)$	Electric field noise
Stark (single dot SO)	δE_{Z}^{z}	Electric field noise
Stark (double dot SO)	Δ (via Δ_{SOC})	Electric field noise
Magnetic gradient $\hat{z}$	δE_{Z}^{z}	Magnetic field noise
Magnetic gradient $\hat{x},\hat{y}$	Δ (via $\delta E_{\text{Z}}^{x,y}$)	Magnetic field noise

Supplementary Table 1. Key noise coupling mechanisms as studied in the accompanying simulations presented in Supplementary Figure 5. These parameters are defined in Hamiltonian equation Supplementary Eq. 1 and include components attributed to electric field such as exchange noise as well as single- and double-dot spin-orbit (SO) effects. Also included are Zeeman energy terms dependent upon magnetic field noise.

SUPPLEMENTARY NOTES

Supplementary Note 1: Extended latching study

As illustrated in Fig. 1e of the main text, the observation of latching of the G2 dot is due to weak coupling to the reservoir[2]. In order to populate the next electron on G2, the existing (1,1) state must co-tunnel via (0,2) where PSB exists. If the state is not blocked (i.e. the S state) then an electron is free to tunnel from the reservoir to fill G1. Otherwise the tunnelling from the reservoir is blocked, resulting in a spin-to-reservoir charge state conversion. State latching is investigated at multiple anti-crossings on the charge stability diagram as shown in Supplementary Figure 1. The anti-crossings attributed to the $N_1 = 1, N_2 = 1-4$ charge configurations are shown with accompanying maps produced in the same method to Fig. 1c (see Ref. [3] and main text). The argument that the latching is indeed the projective mapping of $(0,2)S-(1,1)T \rightarrow (1,2)S-(1,1)T$ is strengthened by the observation of latching only at every second anti-crossing. In both the full stability diagram[4] and maps produced via the three level pulse sequence, strong charge state hysteresis observed[5] under the $G2$ dot after the unloading of the last electron under $G1$ (i.e. $N_1 = 0$), often attributed to the inability for any co-tunnelling of the $G2$ dot via $G1$ to the reservoir. In order to estimate the increase in visibility due to the latched readout, the characteristic relaxation time of the latched state is measured by initializing a random $(1,1)$ state, pulsing rapidly to the Standard-PSB location in $(0,2)$ before pulsing to the latched readout location close to the anti-crossing. The state is taken via $(0,2)$ in order to emulate the measurement protocols used in spin readout experiments. Relaxation of the latched state τ_L is shown in Fig. 3c with a characteristic time of 2.8 ms. Using an optimal measurement integration time of $\tau_M = 0.2$ ms a model for single-shot statistics which accounts for relaxation under τ_L can be implemented[1], yielding a maximum visibility of 98%, by optimizing the threshold current as shown in Fig. 3b. When comparing the maximum visibility of the standard-PSB readout against the latched-PSB process, the increased separation of the readout histograms under latching leads to a reduction in error due to misidentifying a singlet or triplet. The latched-PSB produces a decrease in this misidentification error by nearly 16-fold when compared to the standard-PSB readout process.

Supplementary Note 2: Quantum dot parameters

As discussed in the main text, the system can be described via the same system Hamiltonian presented in Ref.[6], with the addition of the singlet-triplet mixing term. To convert pulsed gate voltages on G1, G2 to detuning $\varepsilon = \mu_2 - \mu_1$, we use the $(0,2)$, $(1,1)$, $(2,0)$ part of the charge stability diagram in Supplementary Figure 1, and model the double dot using the constant interaction model (eq 32 on page 1245 of Ref.[7]). Leaving the unknown average charging energy E_{C} as a free parameter, we self-consistently solve for $\epsilon \sim \alpha_2 V_{\mathrm{G2}} + \alpha_1 V_{\mathrm{G1}}$ and find $\alpha_2 = -6.1 E_{\mathrm{C}}/V$ and $\alpha_1 = 3.7 E_{\mathrm{C}}/V$. Typical charging energies of for this device design is $10-20$ meV, and a value of $E_{\mathrm{C}} = 10$ meV is chosen for ε in these experiments. A transformation of the three level pulse seen in Fig. 1d of the main text is presented in Supplementary Figure 2, allowing for the energy of the first excited state E_{ES} in $G2$ to be measured[3] with respect to E_{C}. This is given by the blockade width, measured as $E_{\mathrm{ES}} = (0.0172 \pm 0.002)E_{\mathrm{C}}$. These values are similar to valley splitting energy E_{VS} measured on previous Si-MOS devices[4,8]. For the latched region the blockade is lifted via the presence of this same excited state, allowing the $G1$ electron to shuttle to $G2$, occupying this excited state.

Supplementary Note 3: Effective Hamiltonian of the $|S_H\rangle$ singlet-triplet subspace

The system Hamiltonian of the singlet-triplet basis[9] can be represented as the following:

$$H\,|\phi\rangle = \begin{bmatrix} \overline{E}_{\mathrm{Z}}^{z} - \varepsilon/2 & 0 & (-\delta E_{\mathrm{Z}}^{x} + i\delta E_{\mathrm{Z}}^{y})/\sqrt{2} & 0 & \Delta_{\mathrm{SOC}} \\ 0 & -\varepsilon/2 & \delta E_{\mathrm{Z}}^{z} & 0 & 0 \\ (-\delta E_{\mathrm{Z}}^{x} - i\delta E_{\mathrm{Z}}^{y})/\sqrt{2} & \delta E_{\mathrm{Z}}^{z} & -\varepsilon/2 & (\delta E_{\mathrm{Z}}^{x} - i\delta E_{\mathrm{Z}}^{y})/\sqrt{2} & t_c(\varepsilon) \\ 0 & 0 & (\delta E_{\mathrm{Z}}^{x} + i\delta E_{\mathrm{Z}}^{y})/\sqrt{2} & -\overline{E}_{\mathrm{Z}}^{z} - \varepsilon/2 & -\Delta_{\mathrm{SOC}} \\ \Delta_{\mathrm{SOC}} & 0 & t_c(\varepsilon) & -\Delta_{\mathrm{SOC}} & \varepsilon/2 \end{bmatrix} \begin{bmatrix} |T_+\rangle \\ |T_0\rangle \\ |(1,1)S\rangle \\ |T_-\rangle \\ |(0,2)S\rangle \end{bmatrix} \quad (1)$$

Here E_{Z}^{n} is a Zeeman energy in given vector direction $\hat{n}$ and is inclusive of the single electron g-factor variability. We can take the liberty of choosing $\hat{z}$ such that the average Zeeman energy in the $\hat{x}$ and $\hat{y}$ directions are zero, leaving only the difference $\delta E_{\mathrm{Z}}^{n}$ as a relevant parameter. In Supplementary Eq. 1, it is evident that there are two sources for singlet-to-triplet transitions: transverse magnetic field gradients ($\delta E_{\mathrm{Z}}^{x,y}$), and a direct spin-flip-transport term Δ_{SOC}

which results from interface spin-orbit coupling. The above Supplementary Eq. 1 is transformed again to represent the hybridized singlet ground state $|S_H\rangle$ and excited state $|G_H\rangle$. By diagonalizing the singlet terms we form this change of basis by introducing $\theta = -\tan^{-1}(2t_c/\varepsilon)$:

$$H|\phi\rangle = \begin{bmatrix} \overline{E}^z_Z - \varepsilon/2 & 0 & 0 & \Delta(\theta) \\ 0 & -\varepsilon/2 & 0 & \delta E^z_Z \cos(\theta) \\ 0 & 0 & -\overline{E}^z_Z - \varepsilon/2 & -\Delta(\theta) \\ \Delta(\theta)^* & \delta E^z_Z \cos(\theta) & -\Delta(\theta)^* & E_{S_H} \end{bmatrix} \begin{bmatrix} |T_+\rangle \\ |T_0\rangle \\ |T_-\rangle \\ |S_H\rangle \end{bmatrix} \tag{2}$$

where we have dropped the higher energy excited state $|G_H\rangle$. The overall coupling term between the hybrid singlet state $|S_H\rangle$ and polarized triplets $|T_\pm\rangle$ is given by the term

$$\Delta(\theta) = -\left(\frac{\delta E^x_Z + i\delta E^y_Z}{\sqrt{2}} \cos(\theta) + \Delta_{\mathrm{SOC}} \sin(\theta) \right). \tag{3}$$

This overall coupling is a combination of spin-orbit effects and magnetic field gradients perpendicular to applied field B^z_0 (such as those attributed to the hyperfine field of a nuclear spin bath).

It is clear that when $\varepsilon \to \infty, \theta \to 0$ and the δE_Z coupling terms dominate in the $(1,1)$, while Δ_{SOC} coupling contributions introduced via $(2S,0)$ are suppressed. Further, the exchange energy $J(\varepsilon) = E_{T_0} - E_{S_H} = -\frac{1}{2}\varepsilon + \frac{1}{2}\sqrt{\varepsilon^2 + 4t^2} \to 0$ and is dependent upon the hybridized singlet energy $E_{S_H} = -\frac{1}{2}\sqrt{\varepsilon^2 + 4t^2} \to E_{T_0}$.

Supplementary Note 4: Landau-Zener excitations

A single-passage Landau-Zener[10] experiment was used to calibrate the control gate voltage ramping rates against the excitation probability[11,12], using the formula $P_{\mathrm{LZ}} = \exp(-2\pi\Delta^2/\hbar\nu)$. The ramping rate directly corresponds to the energy level velocity $\nu = |d(E_{S_H} - E_{T_-})|/dt \gg \Delta^2$ using the device lever arms[13] and the model for exchange coupling $J(\varepsilon)$. The Hamiltonian for modelling these experiments is truncated to include only the hybridized singlet state and $|T_-\rangle$ polarized triplet state, with system described as

$$\begin{bmatrix} \overline{E}_Z - J(\varepsilon) & \Delta(\theta) \\ \Delta(\theta)^* & 0 \end{bmatrix} \begin{bmatrix} |S_H\rangle \\ |T_-\rangle \end{bmatrix}, \tag{4}$$

which is a truncated version of Supplementary Eq. with normalization to the triplet energy E_{T_-}. The energy separation $|d(E_{S_H} - E_{T_-})|$ of this two level system is directly probed by Landau-Zener-Stückelberg interferometry[10,13] as shown in Fig. 2g, and is used to model exchange for these experimental conditions. This shows an asymptotic approach to a Zeeman energy of $E_Z = 4.832$ MHz, corresponding to residual magnetic field $B^z_{OS} = 0.164$ mT. We note that this residual field is of the correct order of magnitude to result from complete nuclear polarization[14] but it may also be due to a residual field from the superconducting solenoid apparatus. For this dataset, $|\Delta(\theta)| = 48.98 \pm 6.27$ kHz is fit.

Illustrated in Fig. 2e of the main text are additional fits of the single passage Landau-Zener experiment. As stated in Supplementary Note , this data was collected using the same device on a different experimental set-up. The magnetic field offset for these datasets was calibrated via a fit to the spin funnel in Fig. 2d, yielding $B^z_{OS} = -1.04 \pm 0.06\pm$ mT. For this residual field, at the location of the minimum energy gap, $|\Delta(\theta)| = 196 \pm 6.3$ kHz. For increased field $B^z_0 = 155$ mT, $|\Delta(\theta)| = 16.72 \pm 1.64$ MHz. The field dependence of this value can be deduced from Supplementary Eq. 3.

Supplementary Note 5: Field dependent data

Spin funnel data used for Hamiltonian parameter fitting is presented in Supplementary Figure 4. All data collated in Fig. 4 of the main text was taken during the same device cool down, using the same experimental set-up for all experiments. The device was transported to a different dilution refrigeration unit (which possessed a magnet capable of reversing polarity), where the full spin funnel data as illustrated in Fig. 2d, and the single passage LZ data from Fig. 2e for both low and high magnetic fields were collected.

Supplementary Note 6: Detuning dependence of tunnel coupling

It has been documented in Si/SiGe based double-dots such as those in Refs.[15,16] that the tunnel coupling can be dependent upon detuning, and can be modelled using the Wentzel-Kramers-Brillouin (WKB) approximation[15]. While full device simulations were not performed here, we make the assumption that a one-dimensional approximation holds[17]. The model used is of a similar form to Ref. [16], giving:

$$t_c(\varepsilon) = \Lambda \left(\sqrt{\exp(2\varphi(\varepsilon)) + 1} - \exp(\varphi(\varepsilon)) \right) \tag{5}$$

where φ is an integral over electron momentum between classical turning points of the 1D potential $\phi(\varepsilon)$[17]. Following the findings in Refs. [15, 16], a phenomenological model for the integral ϕ is simply $\phi = \varepsilon/\zeta$, with ζ numerically fit to 0.59 ± 0.02 meV.

This fit also returns $\Lambda = 4.36 \pm 0.07$ GHz and indicates a tunnel coupling at the anti-crossing of $t_c(\varepsilon = 0) = 1.864 \pm 0.033$ GHz. Using Supplementary Eq. , the data in Fig. 4 was fit using the Hamiltonian described in Eq. 1 of the main text, resulting in fitted value $\delta g = (0.43 \pm 0.02) \times 10^{-3}$.

Magnetic field offsets in low-field experiments as presented in the main text are incorporated in Fig. 4, and are able to tie together the spin-funnel and LZS interferometry data. Remaining variables in this dataset are limited to small shifts in ε for spin-funnel, coherent-exchange and ESR fittings attributed to the separation of preparation point P and $\varepsilon = 0$ which can vary between device calibrations.

Supplementary Note 7: Analysis of experimental errors for exchange pulses

Using the example of the driven exchange oscillations $|\uparrow\downarrow\rangle \leftrightarrow |\downarrow\uparrow\rangle$ illustrated in Fig. 3c,d&e of the main text, we discuss the sources of errors and how they might be mitigated. The first source of error e_{Prep} manifests during the preparation of the $|(0,2)S\rangle$ state, while e_{Meas} refers to the error in conversion from $(0,2)-(1,2)$. The combined error $e_{(0,2)\mathrm{SPAM}}$ is directly observed in the histogram of main text Fig. 1g, measured to be 0.8%.

High fidelity operation of this device requires consideration of the voltage ramp rates. In general, a ramp rate ν_X, for any process X, is the derivative of some energy E with respect to time as a particular anticrossing is traversed. These are then related to an experimentally controlled voltage ramp-rate through the associated function $E(V)$ given by the Hamiltonian model Eq. 1 of the main text. The results we have presented all use linear ramps, enabling simpler analysis of bounds of constant ramp-rates; a future, more sophisticated approach might employ pulse-shaping to accelerate and decelerate voltage ramps to avoid errors.

The first such ramp-rate we consider is ν_{in}, which impacts e_{transfer}, the error of adiabatically transforming $|(0,2)S\rangle$ into $|\uparrow\downarrow\rangle$. To avoid mixing with $|T_-\rangle$, as well as to maintain adiabaticity in the transfer to $|S_H\rangle$, ν_{in} must satisfy $\Delta^2/\hbar \ll \nu_{\mathrm{in}} \ll 4t_c^2(\epsilon)/\hbar$. Further, we require $\nu_{\mathrm{in}} \ll \delta E_Z^2/\hbar$ as detuning ε increases so not as to populate a mixture of $|\downarrow\uparrow\rangle$ and $|\uparrow\downarrow\rangle$. Failure to maintain these conditions may lead to leakage of the desired $|S_H\rangle$ or $|\uparrow\downarrow\rangle$ states into undesired states.

Likewise, the error in conversion from the final state back to the $|(0,2)S\rangle$ state is given by e_{Map}. The resulting state is required to transfer back to the $(0,2)$ charge state, where it can initially mix again based on $\nu_{\mathrm{Out}} \ll (\delta E_{\mathrm{Z}})^2/\hbar$. The $|\downarrow\uparrow\rangle$ state is mapped back to $|(0,2)S\rangle$, where $\Delta^2/\hbar \ll \nu_{\mathrm{Out}} \ll 4t^2(\varepsilon)/\hbar$. For $|\uparrow\downarrow\rangle$, the state is blocked by Pauli-exclusion, and $\nu_{\mathrm{Out}} \ll 2t^2(\varepsilon)/\hbar$ is required near the anti-crossing.

Therefore, constant ramp rates require $\Delta^2/\hbar \ll \nu_{\mathrm{In/Out}} \ll (\delta E_{\mathrm{Z}})^2/\hbar$ to be satisfied to maintain low transfer errors e_{Transfer} and e_{Map}.

The final error mechanism tested is e_{Control}, the error associated with controlling the exchange operation between the ramp in/out stages. After the $(1,1)$ state is prepared, this state is pulsed rapidly back to a point of non-zero exchange coupling at rate ν_{Pulse}. This rate must abide by $\nu_{\mathrm{Pulse}} \gg J^2(\varepsilon)/\hbar$, otherwise a phase error can be introduced based on the integration of exchange during the non-negligible ramping times. Ramping close to the $(1,1)-(0,2)$ transition can also result in leakage to a $(0,2)$ charge state. This is achieved via $\nu_{Pulse} \gg t^2(\varepsilon)/\hbar$.

The above is a very strict set of bounds which could be achieved via shaped pulses. Falling short of this, a trade-off between $e_{\mathrm{Transfer}}/e_{\mathrm{Map}}$ and e_{Control} can be produced by shallower preparation in the $(1,1)$.

For the experimental conditions as presented in the main text, the measurement errors discussed above can be observed in the simulation of state preparation and measurement in Supplementary Figure 5a. Here, the leakage of the prepared state can be observed as both the population of the $|(0,2)S\rangle$ state in the $(1,1)$ region, due to passage through (or in the vicinity of) the $(0,2)-(1,1)$ anti-crossing at $\varepsilon = 0$, and as population of the $T_\pm$ triplet states. The populations illustrated are produced via Monte-Carlo integration over realistic noise parameters for silicon-MOS[15,18], for the pulse sequence shown in Supplementary Figure 5b. Key noise sources include charge noise including gate voltage noise, and magnetic gradient noise. Each noise spectrum can be given by $S(f) = A^2 f_0^{\alpha-1}/f^\alpha$ with $f_0 = 1$ Hz[15]. Magnetic

gradient noise is constructed from measured parameters in literature[15], with $A_m = 0.2$ neV, $\alpha_m = 1.5$. Electric field noise (from charge and voltage fluctuations) is likewise constructed based on literature[18], with $A_e = 4.5$ µeV, $\alpha_c = 1$, including Johnson-Nyquist noise attributed to a 100Ω room temperature resistor.

These two noise sources can couple into the experimental system in various ways, and the key mechanisms studied here are shown in Supplementary Table. 1. Under these noise mechanisms, a simulation which involves a zero-dwell time exchange pulse towards the anti-crossing is shown in Supplementary Figure 5a, with time precision normalized to the associated ramping rates ν_X (as shown by Supplementary Figure 5b). When this preparation, semi-adiabatic transfer, exchange pulse in/out, semi-adiabatic map and readout mechanism are also combined with a dwell time at the exchange point ε, the resulting oscillation characteristics can be fit and compared to the experimental data to Fig. 3e. The resulting visibility V_{Sig}, saturation value V_{Sat} and oscillation decay times T_2^{ρ} can be extracted by fitting with the following $|(0,2)S\rangle$ return probability function:

$$P_S(t) = \frac{V_{\mathrm{Sig}}}{2}\cos(2\pi F t + \vartheta)\exp\left(-\frac{t}{T_2^{\rho}}\right)^2 + V_{\mathrm{Sat}} \tag{6}$$

where t is the dwell time at the exchange point ε. The oscillation frequency indicated in Supplementary Eq. 6 is given by the energy separation of the hybridized singlet and the T_0 triplet state $F = \sqrt{J^2 + \delta E_Z^2}/h$. The variation in this frequency due to the noise sources within the system is given by σ_F and relates to the decay time $T_2^{\rho} = 1/(\pi\sigma_F)$.

The phase term ϑ is close to zero, and is accumulated as an error during state transfer and mapping processes. The key parameters V_{Sig}, V_{Sat} and T_2^{ρ} are shown in Supplementary Figure 5c-e for the dominant noise coupling mechanisms from in Supplementary Table 1. The first key noise coupling mechanism is attributed to electric field fluctuations which couples directly into the exchange term J via ε. A second term is attributed to the magnetic field noise gradients which couple directly into $\delta E_Z^{\hat{z}}$.

The individual simulations for these two key coupling mechanisms are also presented alongside a simulation showing the cumulative noise from all of the mechanisms from Supplementary Table 1 acting together. Comparing the cumulative noise simulation to the individual exchange noise and $\hat{z}$ magnetic gradients shows that the remaining coupling mechanisms from Supplementary Table 1 are suppressed due to coupling via weaker physical effects such as the Stark shift.

These simulated evolutions demonstrate that the two different noise sources have drastically different effects on the experiment. It is observed that the electric field noise components which couple into the system via the exchange $J(\varepsilon)$, lead to a shortening of decay time T_2^{ρ} as shown for shallow ε in Supplementary Figure 5e, while the transfer and mapping processes remain largely unaffected. Under high exchange, the visibility of the oscillations is also degraded due to exponential dependence upon ε.

The other major error source is due to magnetic gradient noise in the $\hat{z}$ direction. Due to direct modulation of the $\delta E_Z^{\hat{z}}$ term, this noise type directly affects the ability to consistently transfer(map) the initial(final) state via the semi-adiabatic ramp. This is made clear from the lifting of V_{Sat} for regions where $J(\varepsilon) \ll \delta E_Z^{\hat{z}}$ in Supplementary Figure 5d. Due to the error in preparation of the $|{\downarrow\uparrow}\rangle$ state, the resulting visibility of oscillations also suffers.

When these noise mechanisms are brought together, the overall simulation resembles that of the data collected for the exchange driven oscillations. The authors note, however, that reasonable trade-off between noise amplitudes A_e and A_m can tweak the overall shape of the visibility curve observed within the cumulative noise simulations. The final point addressed is a discrepancy between simulation visibility compared to data visibility in Supplementary Figure 5c. This is due to the time resolution limitations within the experiment preventing an accurate estimate for the visibility at large exchange. This issue is avoided within the simulations by normalizing the evolution time-step at detuning point ε by the exchange energy J. This approach maintains adequate sampling of the exchange oscillations, facilitating a good fits for Supplementary Eq. 6 return probability envelope.

SUPPLEMENTARY REFERENCES

1 C. Barthel, D. J. Reilly, C. M. Marcus, M. P. Hanson, and A. C. Gossard, "Rapid single-shot measurement of a singlet-triplet qubit," Physical Review Letters **103**, 160503 (2009).

2 P. Harvey-Collard, B. D'Anjou, M. Rudolph, N. T. Jacobson, J. Dominguez, G. A. Ten Eyck, J. R. Wendt, T. Pluym, M. P. Lilly, W. A. Coish, M. Pioro-Ladrière, and M. S. Carroll, "High-fidelity single-shot readout for a spin qubit via an enhanced latching mechanism," Preprint at https://arxiv.org/abs/1703.02651 (2017).

[3] B. M. Maune, M. G. Borselli, B. Huang, T. D. Ladd, P. W. Deelman, K. S. Holabird, A. A. Kiselev, I. Alvarado-Rodriguez, R. S. Ross, A. E. Schmitz, M. Sokolich, C. A. Watson, M. F. Gyure, and A. T. Hunter, "Coherent singlet-triplet oscillations in a silicon-based double quantum dot," Nature **481**, 344–347 (2012).
[4] C. H. Yang, W. H. Lim, F. A. Zwanenburg, and A. S. Dzurak, "Dynamically controlled charge sensing of a few-electron silicon quantum dot," AIP Advances **1**, 042111 (2011).
[5] C. H. Yang, A. Rossi, N. S. Lai, R. Leon, W. H. Lim, and A. S. Dzurak, "Charge state hysteresis in semiconductor quantum dots," Applied Physics Letters **105**, 183505 (2014), arXiv:1407.1625.
[6] M. Veldhorst, C. H. Yang, J. C. C. Hwang, W. Huang, J. P. Dehollain, J. T. Muhonen, S. Simmons, A. Laucht, F. E. Hudson, K. M. Itoh, A. Morello, and A. S. Dzurak, "A two-qubit logic gate in silicon," Nature **526**, 410–414 (2015).
[7] R. Hanson, L. P. Kouwenhoven, J. R. Petta, S. Tarucha, and L. M. K. Vandersypen, "Spins in few-electron quantum dots," Reviews of Modern Physics **79**, 1217–1265 (2007).
[8] J. C. C. Hwang, C. H. Yang, M. Veldhorst, N. Hendrickx, M. A. Fogarty, W. Huang, F. E. Hudson, A. Morello, and A. S. Dzurak, "Impact of g-factors and valleys on spin qubits in a silicon double quantum dot," Physical Review B **96**, 045302 (2017).
[9] J. M. Taylor, J. R. Petta, A. C. Johnson, A. Yacoby, C. M. Marcus, and M. D. Lukin, "Relaxation, dephasing, and quantum control of electron spins in double quantum dots," Physical Review B **76**, 035315 (2007).
[10] S. N. Shevchenko, S. Ashhab, and F. Nori, "Landau Zener Stückelberg interferometry," Physics Reports **492**, 1–30 (2010).
[11] L. D. Landau, "On the theory of transfer of energy at collisions ii," Phys. Z. Sowjetunion **2**, 7 (1932).
[12] C. Zener, "Non-adiabatic crossing of energy levels," Proceedings of the Royal Society of London A: Mathematical, Physical and Engineering Sciences **137**, 696–702 (1932).
[13] J. R. Petta, H. Lu, and A. C. Gossard, "A coherent beam splitter for electronic spin states." Science **327**, 669–672 (2010).
[14] D. J. Reilly, J. M. Taylor, E. A. Laird, J. R. Petta, C. M. Marcus, M. P. Hanson, and A. C. Gossard, "Measurement of temporal correlations of the overhauser field in a double quantum dot," Physical Review Letters **101**, 236803 (2008), arXiv:0712.4033.
[15] K. Eng, T. D. Ladd, A. Smith, M. G. Borselli, A. A. Kiselev, B. H. Fong, K. S. Holabird, T. M. Hazard, B. Huang, P. W. Deelman, I. Milosavljevic, A. E. Schmitz, R. S. Ross, M. F. Gyure, and A. T. Hunter, "Isotopically enhanced triple-quantum-dot qubit," Science Adv. **1**, e1500214 (2015).
[16] M. D. Reed, B. M. Maune, R. W. Andrews, M. G. Borselli, K. Eng, M. P. Jura, A. A. Kiselev, A. E. Schmitz, A. Smith, J. A. Wright, M. F. Gyure, and A. T. Hunter, "Reduced Sensitivity to Charge Noise in Semiconductor Spin Qubits via Symmetric Operation," Physical Review Letters **116**, 110402 (2016).
[17] S. K. Bhattacharya and A. R. P. Rau, "Coulomb spectrum in crossed electric and magnetic fields: Eigenstates of motion in double-minimum potential wells," Physical Review A - Atomic, Molecular, and Optical Physics **26**, 2315–2321 (1982).
[18] J. T. Muhonen, J. P. Dehollain, A. Laucht, F. E. Hudson, R. Kalra, T. Sekiguchi, K. M. Itoh, D. N. Jamieson, J. C. Mccallum, A. S. Dzurak, and A. Morello, "Storing quantum information for 30 seconds in a nanoelectronic device," Nature Nanotechnology **9**, 986–991 (2014).
